# Analysis of carbon emissions from land cover change during 2000 to 2020 in Shandong Province, China

**DOI:** 10.1038/s41598-022-12080-0

**Published:** 2022-05-16

**Authors:** Linye Zhu, Huaqiao Xing, Dongyang Hou

**Affiliations:** 1 School of Surveying and Geo-Informatics, Shandong Jianzhu University, Jinan, Shandong Province China; 2 School of Geosciences and Info-Physics, Central South University, Changsha, China

**Keywords:** Environmental impact, Sustainability

## Abstract

Land cover change affects the carbon emissions of ecosystems in some way. The qualitative and quantitative understanding of carbon emissions from human activities (e.g., land cover change, industrial production, etc.) is highly significant for realizing the objective of carbon neutrality. Therefore, this paper used GlobeLand30 land cover maps, annual average normalised difference vegetation index (NDVI) data, annual average net ecosystem productivity (NEP) data and statistical yearbook data from 2000 to 2020 to explore the relationship between land cover change and carbon emissions. Specifically, it included land cover change, carbon storage changes influenced by land cover change, spatial and temporal analysis of carbon sources and sinks, land use intensity change and anthropogenic carbon emissions. The results of the study show that the main land cover changes in Shandong province during 2000–2020 was cultivated land conversion to artificial surfaces. Among them, the area of cultivated land converted to artificial surfaces from 2000 to 2010 was 4930.62 km^2^, and the proportion of cultivated land converted to artificial surfaces from 2010 to 2020 was as high as 78.35%. The total carbon stock of vegetation affected by land cover change decreased by 463.96 × 10^4^ t and 193.50 × 10^4^ t in 2000–2010 and 2010–2020 respectively. The spatial and temporal distribution of carbon sources and sinks differed more markedly from 2000 to 2020, and land use intensity changes in Shandong Province showed an upward trend. Of the total energy production, industry has the largest energy consumption, followed closely by total energy consumption in transportation, storage and postal services.

## Introduction

Phenomena such as global warming, melting glaciers, rising sea levels and hazy weather indicate that climate change brought about by the greenhouse effect is seriously affecting the future survival of mankind^1–3^. The Earth’s climate is witnessing changes due to the influence of various factors. In recent years, global warming has become a hot issue for research. Emissions of greenhouse gases, e.g., carbon dioxide (CO_2_) emissions, are regarded as a major contributor to global warming^4–6^. Studies have shown that the global average surface temperature has increased by 0.74 °C since the last century^7^, and atmospheric CO_2_ concentrations have increased by up to 1.9 ppm per year^8^. The Global Carbon Budget Project shows that emissions of fossil CO_2_ will increase by 0.5% in 2019, with emissions falling in the US and EU28 and increasing in China, India and the rest of the world^9^. With increasing levels of urbanisation and industrialisation, human activities are emitting large amounts of CO_2_ into the atmosphere. Of these, land cover change is an important contributor to carbon emissions, making up roughly one-third of the carbon emissions generated by human activities from the industrial revolution^10^, contributing to increasing concentrations of CO_2_. Therefore, the study of the interrelationship between land cover change and carbon emissions is of great importance to further reduce carbon emissions.

The implementation of the reform and opening-up policy has led to a significant acceleration of urbanisation in China, most notably the dramatic expansion of artificial surfaces. Land cover change, energy consumption and other related human activities are the sources of carbon emissions and account for a relatively large share. Rapid economic development and high urbanisation levels in China's cities are hotspots for studying carbon emissions caused by land cover change. For example, Deng et al. analysed the influence of expanding construction land on carbon emissions related to energy in China and its provinces from 2001 to 2011^11^. Lu et al. explored the development process and characteristics of regional differences in carbon emissions from agricultural land in 31 provinces in mainland China from 2000 to 2015^12^. Li et al. calculated the 1999–2015 Shanghai energy structure, energy intensity, industrial structure and other factors on carbon emissions from construction land in Shanghai^13^. However, most of the above studies have analysed carbon emissions from a single land cover type, and lack a discussion of carbon emissions from the perspective of changes in different land cover types. Further research scholars have conducted corresponding spatial and temporal analysis of carbon emissions for the different land cover types. For example, Shi et al. assessed the spatio-temporal distribution of urban CO_2_ emissions in China from 1997 to 2012 at national to regional to urban cluster scales^14^. Zhu et al. explored changes in vegetation carbon storage and soil organic carbon storage due to land cover change in Zhejiang Province between 1970 and 2010^7^. Zhang et al. analysed carbon emissions in the Yellow River Delta region from 2000 to 2019 in terms of spatial and temporal distribution^15^. However, the aforementioned papers mainly focused on analysing the spatial and temporal distribution of carbon emissions, and relatively few studies have analysed land cover change, carbon emissions from land cover change, land use intensity change, and the distribution of carbon sources and sinks from multiple perspectives. Therefore, exploring land cover change and its carbon emission impact from an integrated perspective is of theoretical significance in formulating scientific and effective low-carbon land use planning to achieve the goal of carbon neutrality.

On 15 September 2020, the 30 m Global Land Cover 2020 remote sensing mapping data was released. The GlobeLand30 data consists of 10 primary types, e.g., cultivated land, forest, grassland, shrub land, wetland, water bodies, artificial surfaces, bare land, etc^16^. The overall accuracy of the 2010 GlobeLand30 data is 83.50% with the kappa coefficient of 0.78^17^. The overall accuracy of the 2020 GlobeLand30 data is 85.72% with the kappa coefficient of 0.82. The release of GlobeLand30 provides a data base for large-scale land cover change studies, and has been used by various scholars for studies on a large regional scale^18,19^.

According to the China Emission Accounts and Datasets (CEADs), Shandong Province ranks among the top five provinces in China in terms of total carbon emissions, and is a province with both high total carbon and high carbon intensity^20,21^, which puts great pressure on carbon control efforts. Therefore, Shandong Province can be used as a typical province for regional carbon emissions, and it is of great practical importance to study the carbon emissions from land cover changes in Shandong Province over the last 20 years.

Therefore, this paper takes Shandong Province as the research object and focuses on four aspects, i.e., land cover change, land cover-influenced carbon storage change, spatial–temporal distribution of carbon sources and sinks, land use intensity change and anthropogenic carbon emissions. Firstly, the amount, rate of change and transfer matrix were explored for land cover using GlobeLand30 maps for 2000, 2010 and 2020. Secondly, carbon stock changes affected by land cover change were analysed according to land cover type. Then, annual average normalised difference vegetation index (NDVI) data calculated using remote sensing images and annual average net ecosystem productivity (NEP) data were used for spatial and temporal analysis of carbon sources and sinks. Finally, land use intensity change and anthropogenic carbon emissions were explored with statistical yearbook data, and the above changes were analysed for attribution and recommendations were made accordingly.

The purpose of this paper is to analyse the interactions between land cover change and carbon emissions in Shandong Province from multiple perspectives, and to use the latest land cover data to explore land cover change and carbon emissions in a rational and effective way. The contributions of this paper are as follows. (1) This study provides a comprehensive analysis of land cover change, land use intensity change, and the spatial and temporal distribution of carbon sources and sinks in Shandong Province. (2) This study explores the changes in vegetation carbon storage due to land cover change and anthropogenic carbon emissions in Shandong Province. (3) This study provides an attribution analysis of the above-mentioned land cover changes and carbon emissions and proposes corresponding solutions. This study is crucial for the development of low carbon land management policies.

## Materials and methods

**Figure 1:**
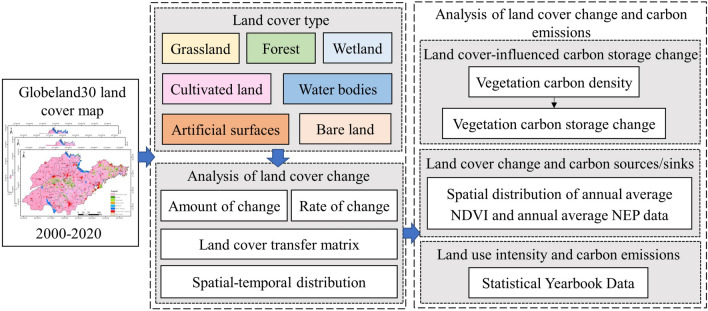
Flowchart of this study.

Figure 1 shows the framework of this study. Firstly, the GlobeLand30 land cover maps for 2000, 2010 and 2020 were used to calculate the amount of change, the rate of change and the transfer matrix for the study period. Secondly, vegetation carbon density was calculated based on land cover type changes to obtain the changes in carbon storage influenced by land cover change. Then, spatial and temporal analysis of carbon sources and sinks were carried out using annual average NDVI data and annual average NEP data. Finally, land use intensity changes were defined with statistical yearbook data, land use intensity change and anthropogenic carbon emissions were analysed, corresponding attribution analysis was carried out and recommendations were provided.

### Study area

Shandong Province is located in the eastern coastal region of China (34° 22.9′–38° 24.01′ N, 114° 47.5′–122° 42.3′ E)^22^. According to the National Bureau of Statistics, Shandong Province ranks third in GDP and is an important economic province in China with a GDP of 7312.9 billion Yuan in 2020. The resident population of Shandong Province is 101,527,500 in 2020. Due to the significant increase in population and economy, the artificial surfaces are rising rapidly and carbon emissions are growing extremely fast^23^.

### Data

The data used in this study were land cover maps, NDVI data and socio-economic data. The land cover maps were GlobeLand30 data for 2000, 2010 and 2020, which were used to explore land cover changes in Shandong Province. Annual average NDVI data were obtained by coding and calculating remote sensing images on the Google Earth Engine platform (https://code.earthengine.google.com/)^24,25^ as vegetation growth state change data. The global day-by-day NEP simulation data product^26–28^ was provided by National Ecosystem Science Data Center, National Science & Technology Infrastructure of China (http://www.nesdc.org.cn). Annual average NEP data for 2000, 2010 and 2019 were obtained by calculating global day-by-day NEP simulation data. Socio-economic data were derived from the Shandong Provincial Statistical Yearbook to obtain the land use intensity changes and energy-related consumption. Where land use intensity indicates the intensity of human activities.

### Methods

#### Land cover change index

The land cover change index is used to calculate the magnitude of change for each land cover type in different years. The calculation equation is as follows.

1 $$ LCI = \frac{{A_{b} - A_{a} }}{{A_{a} }} \times 100\% $$

where $$A_{a}$$ and $$A_{b}$$ represent the area of a particular land cover type in year $$a $$ and year $$b$$ respectively.

#### Normalised difference vegetation index

The NDVI^29,30^ is a good indicator of crop growth and nutrient information and is useful for checking vegetation growth status and vegetation cover. The NDVI equation is given below.

2 $$ NDVI = \frac{NIR - RED}{{NIR + RED}} $$

where $NIR$ and $RED$ indicates the near-infrared band and red band, respectively.

#### Land cover change transfer matrix

The land cover change transfer matrix^31,32^ is a two-dimensional matrix containing quantitative relationships between the inter-transformation of land cover type data calculated for different time periods within the same study area, allowing visualisation of the transitions between land cover types. The land cover change transfer matrix was obtained by calculating GlobeLand30 data for 2000–2020 using ArcGIS 10.2 software.

#### Land cover change-vegetation carbon storage change model

The land cover change-vegetation carbon storage change model was applied to calculate the change in vegetation carbon storage due to land cover type change^33^. Based on the land cover change transfer matrix of Shandong Province and the vegetation carbon density data of each land cover type, the corresponding vegetation carbon density was assigned to the land cover change data using ArcGIS10.2. The vegetation carbon storage change caused by the land cover type change was calculated by the following equation. The vegetation carbon density data are shown in Table 1, and were taken from reference^7,34^. Changes in soil organic carbon due to land cover type change were not considered because of the long time period over which changes in soil organic carbon occurred^35^. The vegetation carbon storage equation is given below.

3 $$ \Delta C_{ij} = \left( {V_{i} - V_{j} } \right) \times A_{ij} $$

where $\Delta C_{ij}$ is the change in vegetation carbon storage caused by the conversion of land cover type from $i$ to $j$; $V_{i}$ and $V_{j}$ are the vegetation carbon density of land cover types $i$ and $j$, respectively; $A_{ij}$ is the area of land cover type converted from $i$ to $j$.

**Table 1:** Carbon density of vegetation in different land cover types.

Land cover type	Vegetation carbon density (t C/ha)		
	X	X_min_	X_max_
Cultivated land	3.25	1.29	5.70
Forest	28.11	12.06	50.18
Grassland	1.24	0.00	2.30
Wetland	0.67	0.00	1.80
Water bodies	–	–	–
Artificial surfaces	–	–	–
Bare land	0.67	0.00	1.80

## Results

### Carbon storage change arising from land cover change

**Figure 2:**
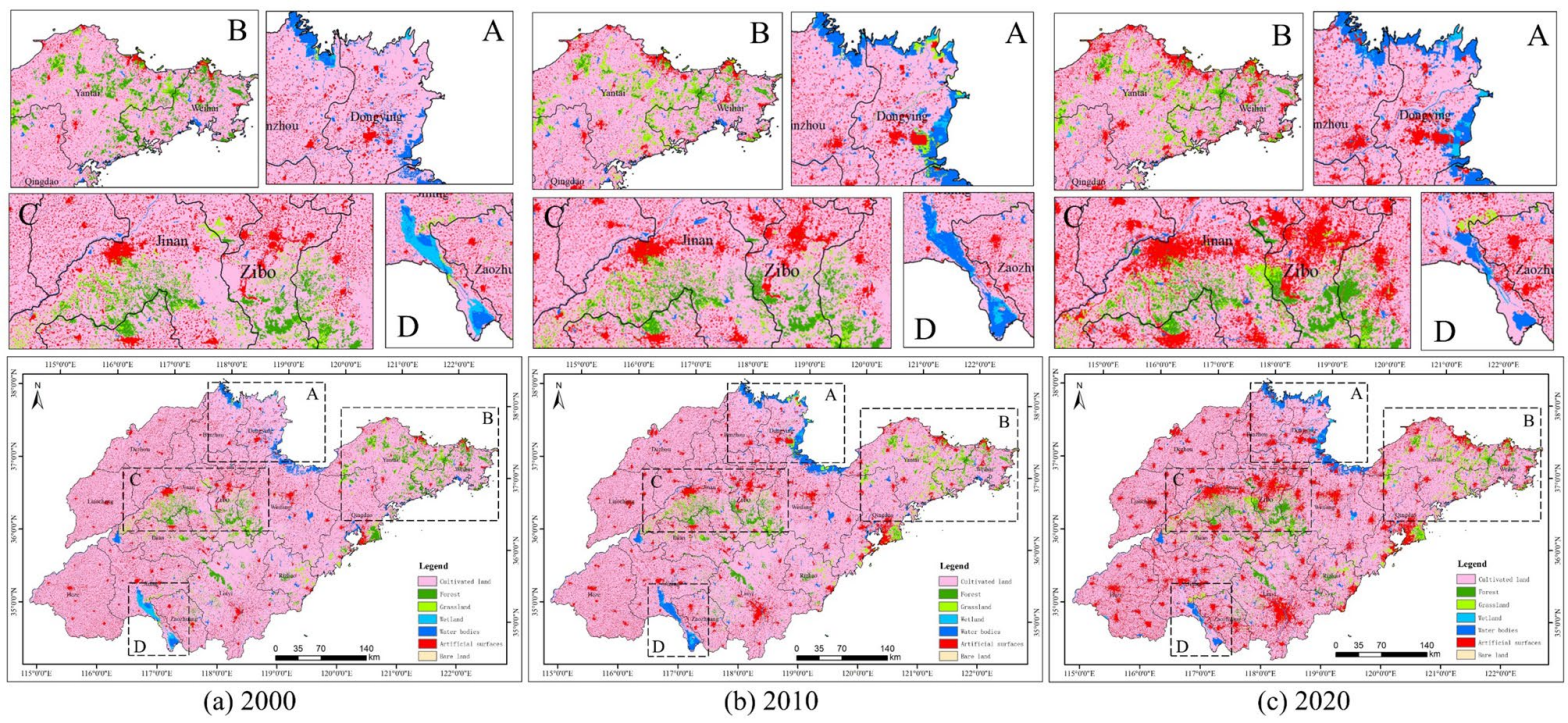
Spatial pattern of land cover in Shandong Province.

As shown in Fig. 2, Shandong Province had the largest share of cultivated land, but the area of cultivated land declined from 126,369.11 km^2^ in 2000 to 111,661.83 km^2^ in 2020 (Table 2). Forest showed a trend of decrease followed by increase, decreasing by 25.42% from 2000 to 2010 and increasing by 13.21% from 2010 to 2020. Grassland tended to expand, with an increase of 33.96% between 2000 and 2010. Wetland and bare land both accounted for a relatively small proportion of the area and did not change much. Water bodies increased by 1467.85 km^2^ between 2000 and 2010, the rate of change up to 41.63%, and dropped by 375.72 km^2^ between 2010 and 2020. The growth in artificial surfaces was particularly rapid during this 20-year period, with an increase in area of 12,562.73 km^2^.

**Table 2:** Land cover change in Shandong Province, 2000–2020.

Year	Change	Land cover type						
		Cultivated land	Forest	Grassland	Wetland	Water bodies	Artificial surfaces	Bare land
2000	Area (km^2^)	126,369.11	4922.72	2733.08	775.14	3525.82	15,524.59	126.28
	Proportion (%)	82.07	3.20	1.78	0.50	2.29	10.08	0.08
2010	Area (km^2^)	122,607.39	3671.28	3661.24	548.86	4993.67	18,324.18	158.41
	Proportion (%)	79.63	2.39	2.38	0.36	3.24	11.90	0.10
2020	Area (km^2^)	111,661.83	4156.20	4019.51	625.95	4617.95	28,087.32	114.87
	Proportion (%)	72.85	2.72	2.62	0.41	3.01	18.32	0.07
2000–2010	Amount of change (km^2^)	− 3761.72	− 1251.44	928.16	− 226.28	1467.85	2799.59	32.13
	Rate of change (%)	− 2.98	− 25.42	33.96	− 29.19	41.63	18.03	25.44
2010–2020	Amount of change (km^2^)	− 10,945.56	484.92	358.27	77.09	− 375.72	9763.14	− 43.54
	Rate of change (%)	− 8.93	13.21	9.79	14.05	− 7.52	53.28	− 27.49

**Figure 3:**
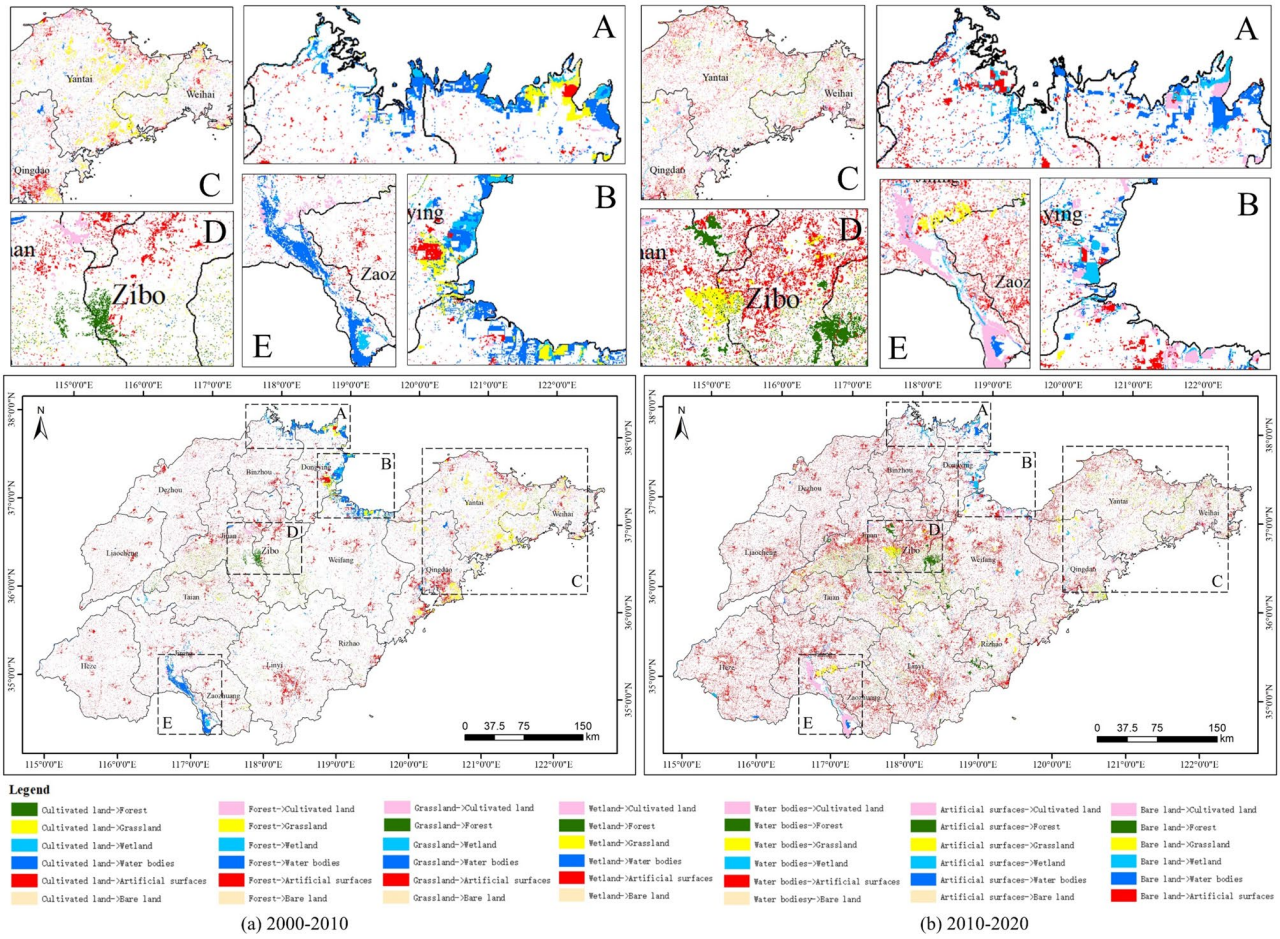
Spatial patterns of land cover transfer matrix in Shandong Province.

Figure 3 shows that, generally speaking, the most pronounced land type change that occurred in Shandong Province was the conversion of cultivated land into artificial surfaces. As shown in Table 3, from 2000 to 2010, the total area of cultivated land transferred out was 7748.14 km^2^, of which 4930.62 km^2^ (63.64%) was transferred to artificial surfaces, while some cultivated land was converted to other land types such as forest and grassland. The conversion of cultivated land to artificial surfaces from 2010 to 2020 accounted for 78.35%. As can be seen, the level of urbanisation in Shandong Province has continued to rise during this 20-year period.

**Table 3:** Land cover transfer matrix for 2000–2020.

Area (km^2^)	Cultivated land	Forest	Grassland	Wetland	Water bodies	Artificial surfaces	Bare land	Total 2010
Cultivated land	118,705.79	663.15	359.64	100.01	575.44	2281.61	5.63	122,691.27
Forest	278.87	3130.35	228.30	1.00	2.87	8.93	21.52	3671.84
Grassland	547.12	1054.69	1958.87	9.80	34.92	26.10	13.71	3645.21
Wetland	272.05	0.06	7.59	171.39	93.76	1.56	0.08	546.49
Water bodies	1684.48	14.57	64.91	467.81	2702.04	22.19	12.29	4968.29
Artificial surfaces	4930.62	38.00	86.69	8.32	68.97	13,132.23	0.93	18,265.76
Bare land	35.00	23.29	9.04	2.59	16.23	0.24	67.32	153.71
Total 2000	126,453.93	4924.11	2715.04	760.92	3494.23	15,472.86	121.48	153,942.57

Area (km^2^)	Cultivated land	Forest	Grassland	Wetland	Water bodies	Artificial surfaces	Bare land	Total 2020
Cultivated land	106,619.90	454.20	528.93	154.42	1455.77	3020.75	26.07	112,260.04
Forest	1000.17	2734.88	393.31	0.15	2.85	13.58	26.67	4171.61
Grassland	1167.07	396.18	2346.70	1.36	34.21	50.78	13.73	4010.03
Wetland	118.92	0.21	88.95	163.48	228.08	5.62	15.32	620.58
Water bodies	1162.41	6.11	133.30	209.65	3007.86	58.92	35.57	4613.82
Artificial surfaces	12,588.96	54.13	132.04	15.40	238.30	15,118.13	5.40	28,152.36
Bare land	30.33	25.59	19.42	0.07	3.37	0.81	30.94	110.53
Total 2010	122,687.76	3671.30	3642.65	544.53	4970.44	18,268.59	153.70	153,938.97

Different land cover types have different vegetation carbon densities. Cultivated land is 325 t C/km^2^, forest is 2811 t C/km^2^, grassland is 124 t C/km^2^, wetland and bare land is 67 t C/km^2^, while artificial surfaces and water bodies have no vegetation carbon density. As a result of land cover change, the total carbon stock of vegetation reduced by 463.96 × 10^4^ t and 193.50 × 10^4^ t between 2000–2010 and 2010–2020, respectively. As shown in Table 4, the decrease in total vegetation carbon storage from 2000 to 2010 was mainly due to the conversion of cultivated land and forest to other land, which decreased by 164.59 × 10^4^ t and 469.44 × 10^4^ t respectively. In particular, the conversion of forest to grassland vegetation carbon storage reduced by 283.39 × 10^4^ t, and the conversion of cultivated land to artificial surfaces decreased by 160.25 × 10^4^ t. Other land types converted to water bodies also resulted in some degree of carbon loss, totalling 105.65 × 10^4^ t between 2000 and 2020. Therefore, the main factors affecting the change in vegetation carbon storage were the reduction of cultivated land and forest.

**Table 4:** Carbon storage change transfer matrix 2000–2020.

Carbon storage/10^4^ t	Cultivated land	Forest	Grassland	Wetland	Water bodies	Artificial surfaces	Bare land	Total 2010
Cultivated land	0.00	− 164.86	7.23	2.58	18.70	74.15	0.15	− 62.05
Forest	69.33	0.00	61.35	0.28	0.81	2.51	5.91	140.19
Grassland	− 11.00	− 283.39	0.00	0.06	0.43	0.32	0.08	− 293.50
Wetland	− 7.02	− 0.02	− 0.04	0.00	0.63	0.01	0.00	− 6.44
Water bodies	− 54.75	− 4.10	− 0.80	− 3.13	0.00	0.00	− 0.08	− 62.86
Artificial surfaces	− 160.25	− 10.68	− 1.07	− 0.06	0.00	0.00	− 0.01	− 172.07
Bare land	− 0.90	− 6.39	− 0.05	0.00	0.11	0.00	0.00	− 7.23
Total 2000	− 164.59	− 469.44	66.62	− 0.27	20.68	76.99	6.05	− 463.96

Carbon storage/10^4^ t	Cultivated land	Forest	Grassland	Wetland	Water bodies	Artificial surfaces	Bare land	Total 2020
Cultivated land	0.00	− 112.91	10.63	3.98	47.31	98.17	0.67	47.85
Forest	248.64	0.00	105.68	0.04	0.80	3.82	7.32	366.30
Grassland	− 23.46	− 106.45	0.00	0.01	0.42	0.63	0.08	− 128.77
Wetland	− 3.07	− 0.06	− 0.51	0.00	1.53	0.04	0.00	− 2.07
Water bodies	− 37.78	− 1.72	− 1.65	− 1.40	0.00	0.00	− 0.24	− 42.79
Artificial surfaces	− 409.14	− 15.22	− 1.64	− 0.10	0.00	0.00	− 0.04	− 426.14
Bare land	− 0.78	− 7.02	− 0.11	0.00	0.02	0.01	0.00	− 7.88
Total 2010	− 225.59	− 243.38	112.4	2.53	50.08	102.67	7.79	− 193.50

**Figure 4:**
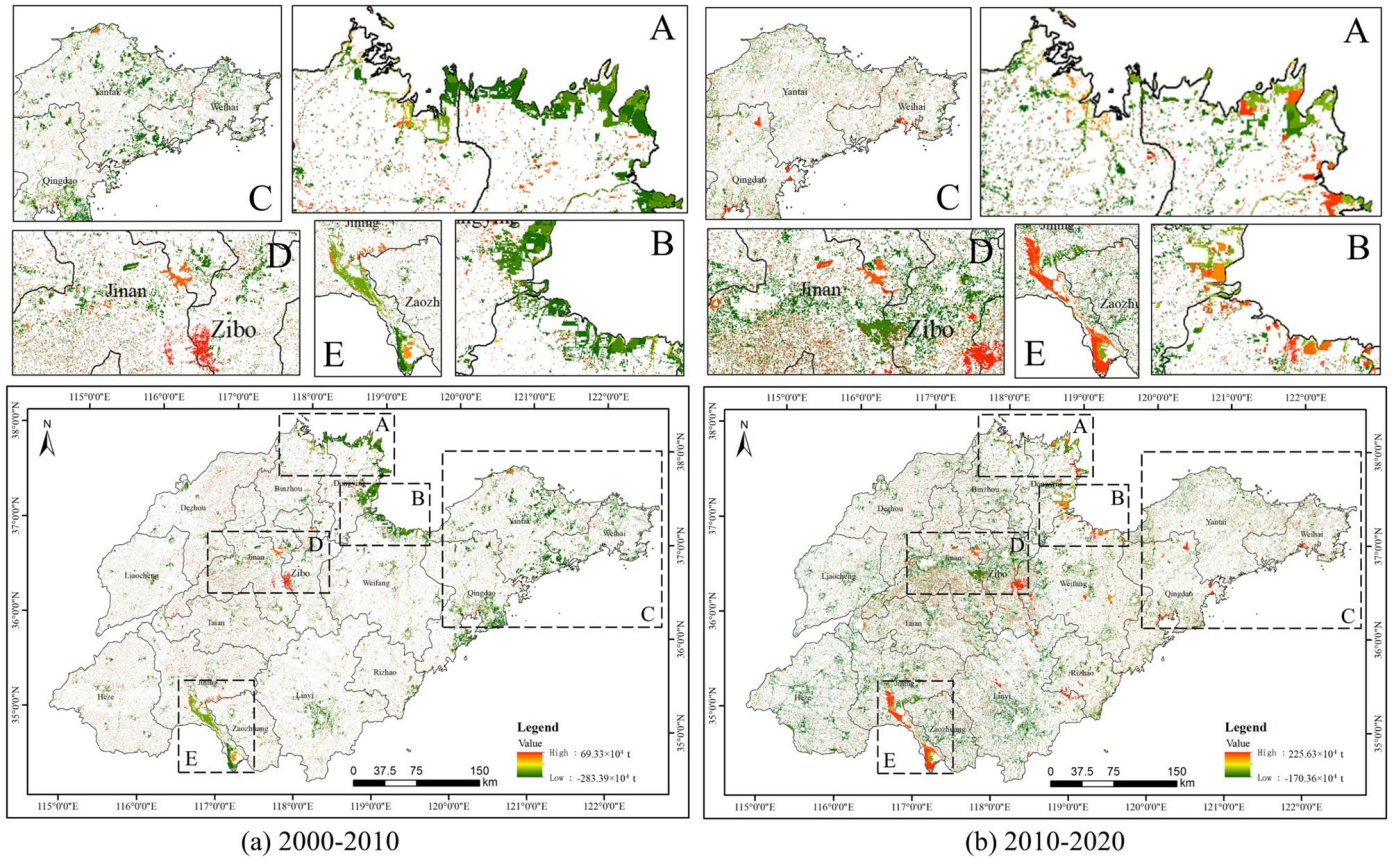
Spatial patterns of vegetation carbon storage in Shandong Province.

Figure 4 shows the change of vegetation carbon storage in Shandong Province from 2000 to 2020. Between 2000 and 2010, there was a significant decrease in the northern coastal areas (e.g., Dongying City, Weifang City) and southern areas (e.g., Jining City) of Shandong Province. More significant decreases also existed in Yantai City, Weihai City, Qingdao City and Linyi City. The central part of Shandong Province (e.g., Jinan City, Zibo City) experienced a more pronounced gain in vegetation carbon storage. Other regions were more fragmented, but still presented mainly declines. During the period 2010–2020, Linyi City, Jinan City and Zibo City had more obvious decreases in vegetation carbon storage, and were largely concentrated in areas with expanding artificial surfaces. Dongying City, Weifang City and Jining City were the main areas where vegetation carbon storage increased. The areas of change in vegetation carbon storage varied considerably over this 20-year period.

### Carbon sources and carbon sinks

**Figure 5:**
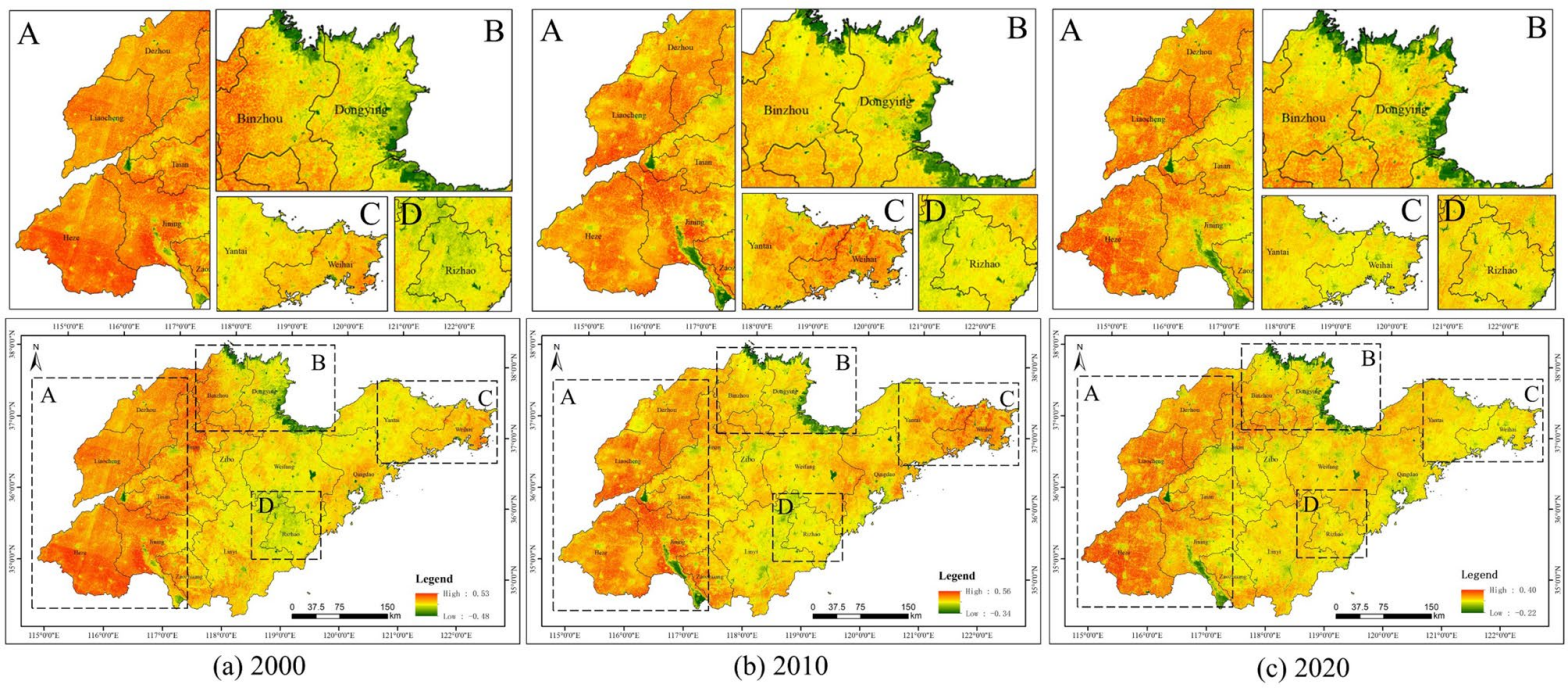
Spatial patterns of annual average NDVI in Shandong Province.

**Figure 6:**
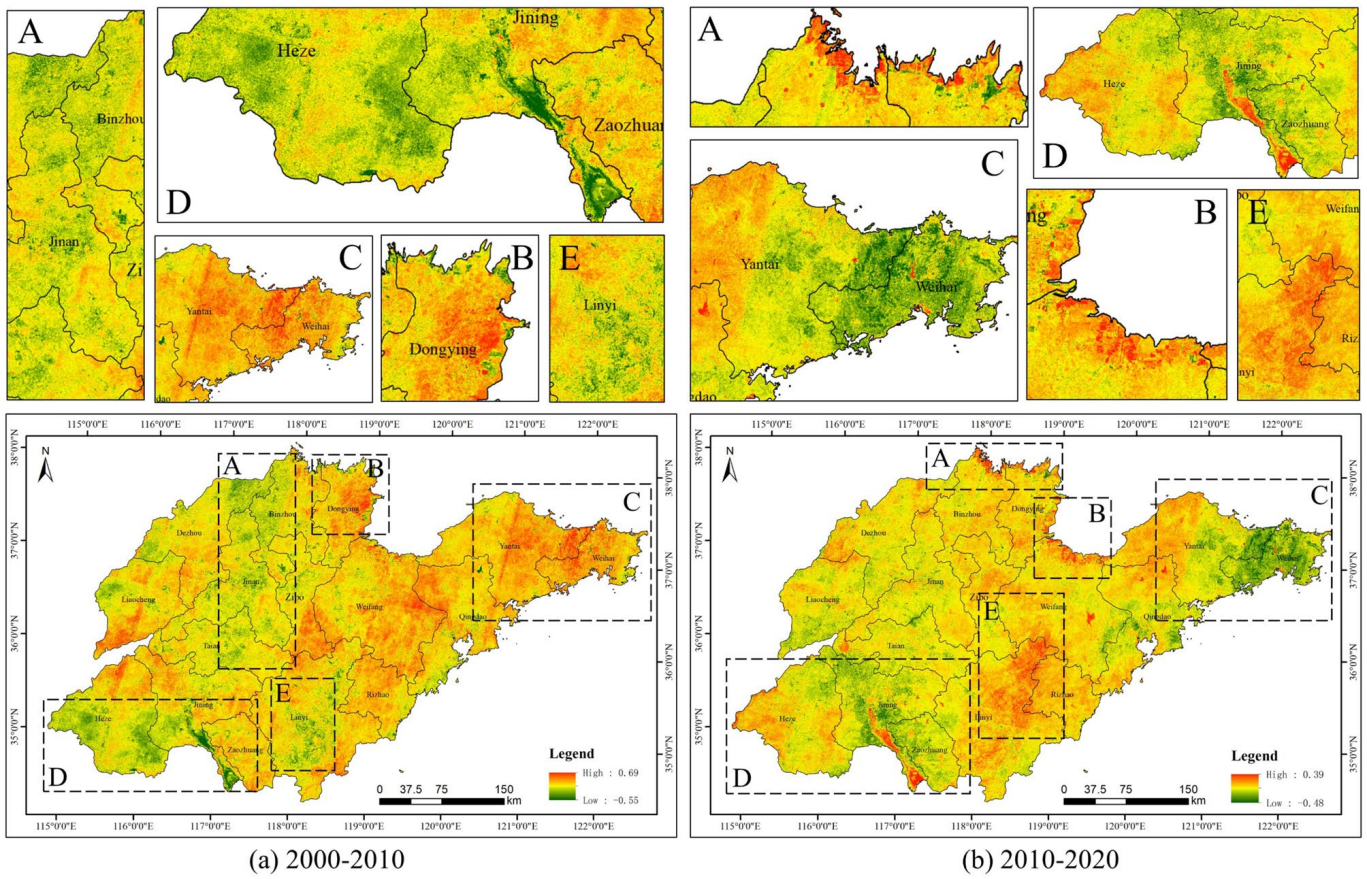
Spatial patterns of annual average NDVI change in Shandong Province.

Figure 5 presents the spatial distribution of the annual average NDVI in Shandong Province from 2000 to 2020, which ranges from − 0.48 to 0.56. Areas with higher NDVI values were located in the western and northeastern parts of Shandong Province, while areas with lower NDVI values were concentrated in the northern coastal areas of Shandong Province, the Nansi Lake region and Rizhao City. Most of the low annual average NDVI values were focused on watersheds. As shown in Fig. 6, Yantai City, Weihai City, Weifang City and Dongying City experienced a dramatic increase in annual average NDVI values between 2000 and 2010. Central Linyi City, southern Jining City, southern Heze City, northern Dezhou City and western Binzhou City displayed a more pronounced decrease in annual average NDVI. From 2010 to 2020, noticeable decreases of annual average NDVI existed in Weihai City, Yantai City, Jining City, Zaozhuang City and Qingdao City. The annual average NDVI rose significantly in Linyi City and Nansi Lake region.

**Figure 7:**
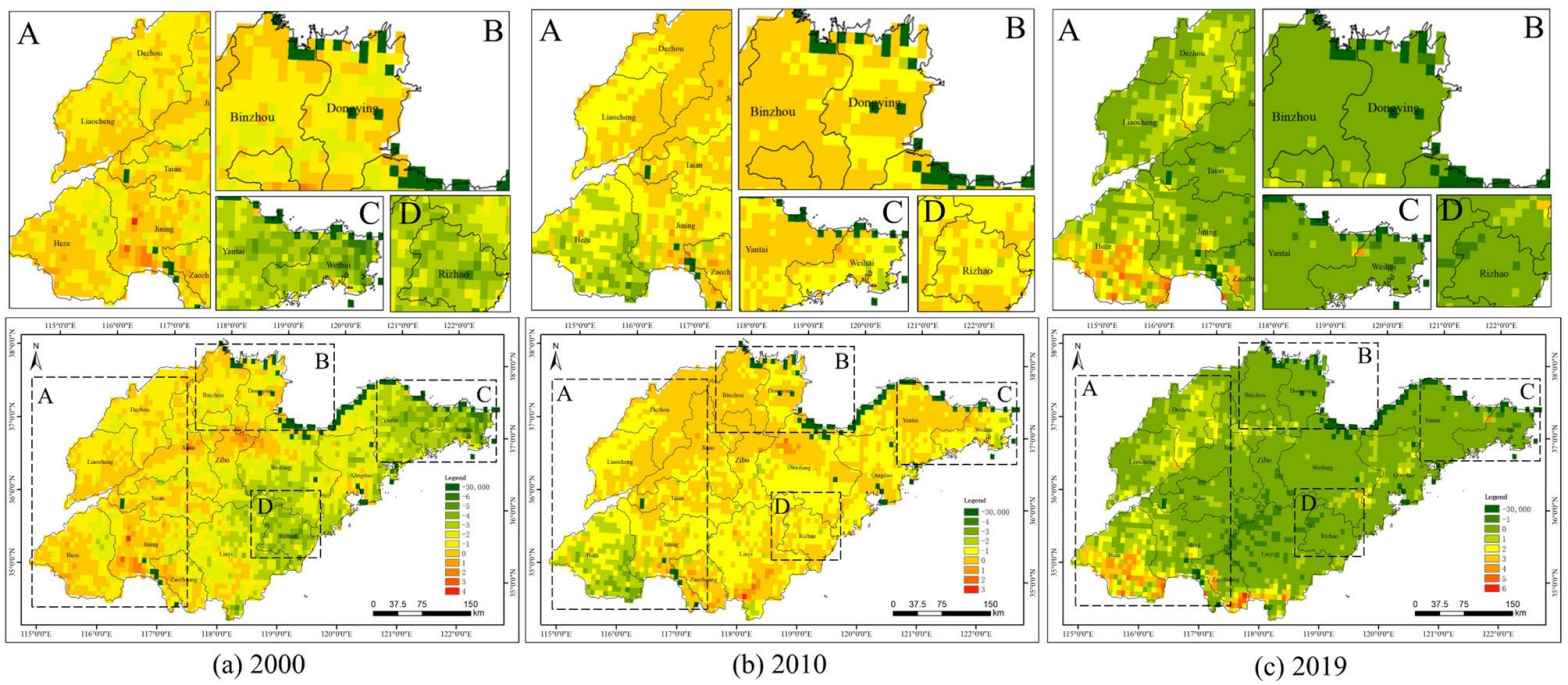
Spatial patterns of annual average NEP in Shandong Province.

**Figure 8:**
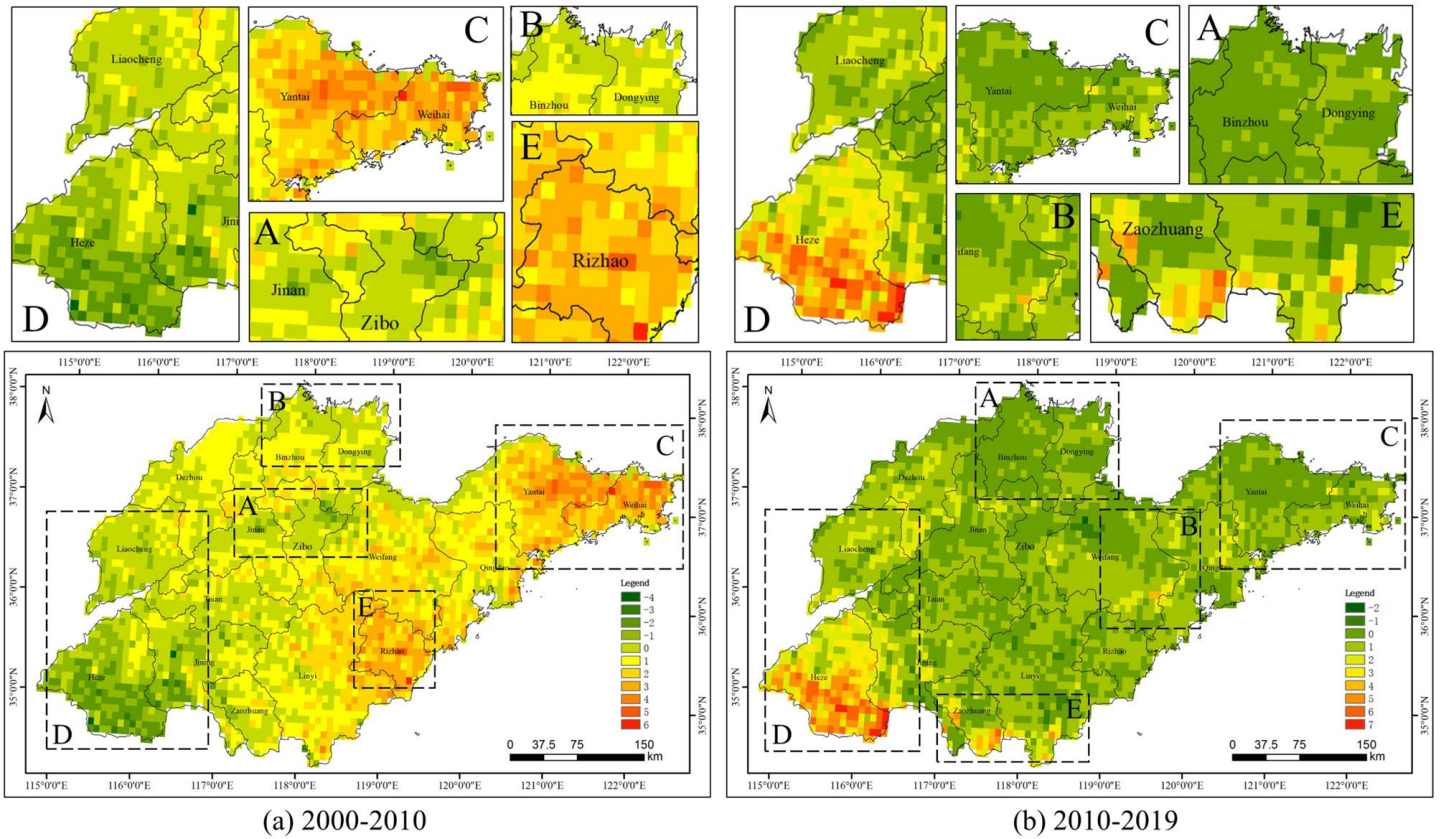
Spatial patterns of annual average NEP change in Shandong Province.

Figure 7 shows the annual average NEP data for Shandong Province from 2000 to 2019. The distribution of carbon sources (negative areas) in 2000 was relatively wide, and carbon sources in 2000 were largely located in the northeastern part of Shandong Province (i.e., Yantai City and Weihai City), Rizhao City and its surrounding cities. Carbon sinks (positive areas) in 2000 were mainly located in Zibo City, Jinan City, Jining City and Heze City. In 2010, carbon sources were mainly located in the southwestern part of Shandong Province, and carbon sinks were mainly located in the southern part of Linyi City and the northwestern part of Weifang City. Carbon sources in 2019 were mainly concentrated in the south-central region of Shandong Province, and carbon sinks were mainly located in the western and south-western regions of Shandong Province. As shown in Fig. 8, the NEP in southwestern Shandong Province and near Zibo City declined significantly from 2000 to 2010, while the NEP in other regions basically showed an increasing trend. The NEP in all regions of Shandong Province showed different degrees of increase from 2010 to 2019.

### Land use intensity change and carbon emissions

According to the data from Shandong Statistical Yearbook, urban population, GDP, industrial output value, agricultural output value, construction output value, investment in fixed assets, shipments quantity and electricity consumption were used as land use intensity changes indicators, as shown in Table 5. As the Statistical Yearbook does not yet have data for 2020, data for 2019 was appiled for comparison. The urban population grew steadily, with GDP increasing from 8278.06 × 10^8^ Yuan in 2000 to 71,067.53 × 10^8^ Yuan in 2019. Industrial output value, agricultural output value, and construction output value have also seen more significant growth, especially from 2000 to 2010, with industrial output value and construction output value increasing by 4.27 times and 4.57 times respectively. Investment in fixed assets increased by a factor of 9.15 between 2000 and 2010. Shipments quantity rose from 92,483 × 10^4^ t in 2000 to 304,732 × 10^4^ t in 2019. Electricity consumption increased significantly between 2000 and 2019, transforming from 1000.49 × 10^8^ to 6218.70 × 10^8^ kwh.

**Table 5:** Indicators related to land use intensity change.

Year	Urban population	GDP	Industrial output value	Agricultural output value	Construction output value	Investment in fixed assets	Shipments quantity	Electricity consumption
Unit	10,000 people	100,000,000 Yuan					10,000 t	100,000,000 kwh
2000	2409	8278.06	3620.06	1300.44	500.13	2542.65	92,483	1000.49
2010	3839	33,922.49	15,449.95	3670.07	2283.13	23,276.69	298,055	3298.46
2019	5068	71,067.53	22,985.13	4914.43	5441.91	–	304,732	6218.70
Interval	Increasing rate							
2000–2010	1.59	4.10	4.27	2.82	4.57	9.15	3.22	3.30
2010–2019	1.32	2.09	1.49	1.34	2.38	0.00	1.02	1.89

As shown in Table 6, total energy production in Shandong Province shifted from 9648.75 × 10^4^ t in 2000 to 12,539.10 × 10^4^ t in 2019. Total energy consumption went from 34,323.00 × 10^4^ t in 2010 to 41,390.00 × 10^4^ t in 2019. In particular, industry accounted for the largest share of total energy consumption, accounting for about 75.60–78.69%, followed by total energy consumption in the transportation, storage and postal services. Total energy consumption for domestic consumption showed a clear upward trend. Total energy consumption in the construction industry saw a more significant decline. Total energy consumption of agriculture, forestry, fisheries, wholesale and retail trade, and accommodation and catering, although also increased, remained a relatively small share of the total. Total energy consumption in other sectors also exhibited highly remarkable growth.

**Table 6:** Energy consumption in different sectors.

Year	Total energy production	Total energy consumption	Total energy consumption in agriculture, forestry and fisheries	Total energy consumption in industry	Total energy consumption in the construction industry	Total energy consumption in the transportation, storage and postal services	Total energy consumption in wholesale and retail trade, accommodation and catering	Total energy consumption in other sectors	Total energy consumption for domestic consumption
Unit	10,000 t SCE								
2000	9648.75	–	–	–	–	–	–	–	–
2010	16,055.71	34,323.00	389.00	27,010.00	663.00	2458.00	937.00	876.00	1990.00
2019	12,539.10	41,390.00	599.70	31,293.70	481.40	2381.80	986.70	1454.40	4192.40

## Discussion

### Attribution analysis of land cover change and carbon emission change

The conversion of cultivated land to artificial surfaces and the conversion of forest to cultivated land and grassland in Shandong Province are the main reasons for the reduction of carbon storage. This is a very common phenomenon for the current urbanisation process in China. Economic growth and population increase are the main factors leading to the change of artificial surfaces^36^. Although Shandong Province has promulgated relevant policies to protect cultivated land, due to the increasing conflict between artificial surfaces and cultivated land, the policy of protecting cultivated land has not been able to stop the expansion of artificial surfaces, especially in areas with better economic development (e.g., Qingdao City, Jinan City)^37,38^.

The conversion of water bodies and cultivated land is mainly concentrated in Jining City, which is also the region where changes in vegetation carbon storage are more pronounced. Jining City covers Weishan Lake, the largest freshwater lake in northern China, and the construction of water storage facilities such as dams and river embankments, as well as river ditches on agricultural land and aquaculture ponds, has led to large changes in the area of water bodies in Jining City^39,40^. In addition, there may be large variations in climate between the years chosen to generate GlobeLand30 data. Therefore, there may be errors in the interpretation of the GlobeLand30 data, resulting in changes in the area of water bodies.

The change in water bodies and wetland conversion is mainly distributed in Dongying City. Dongying City is located at the mouth of the Yellow River and has a low, flat topography that makes drainage difficult and is influenced by the amount of water in the Yellow River, making it easy to form wetlands^41,42^. The influence of policy factors is the main cause of wetland change in water bodies in Shandong Province. The promulgation of relevant policies such as the Measures for the Protection of Wetlands in Shandong Province and the implementation of wetland protection subsidies, together with the unique geographical advantages of Shandong Province, have promoted the generation and expansion of wetlands.

The spatial and temporal distribution of carbon sources and sinks in Shandong province differs greatly between 2000 and 2019. According to land use intensity change and carbon emissions, it can be observed that population increase and economic development have led to a rapid rise in total energy consumption and a marked growth in land use intensity change, which in turn generates large amounts of carbon emissions, especially from industrial production land.

### Recommendations related to land planning and carbon emissions

With the above analysis, it is necessary to reduce carbon emissions scientifically and effectively and to increase the carbon storage capacity of vegetation. Increasing the vegetation cover of artificial surfaces is an effective way to offset carbon losses, with forest having the highest vegetation carbon density. Planting high biomass vegetation on artificial surfaces can be a valuable method to increase carbon storage.

Furthermore, the conservation of land types such as wetland, grassland and forest is also an useful channel to increase the amount of carbon stored in vegetation. Combined with relevant conservation policies, the establishment of wetland nature conservation parks and forest conservation parks, and the improvement of relevant legal systems for environmental protection, such as the Regulations on the Return of Cultivated land to Forests^43,44^, promote the increase of forest, grassland and wetland areas.

On this basis, carbon emissions should be reduced artificially in all aspects of life, from daily life to industrial production. In industrial production, try to use clean energy and renewable energy to gradually replace fossil energy^45^. In agricultural cultivation, improve the utilisation of fertilisers and reduce fertiliser consumption while ensuring crop yields^46^. In daily life, reduce energy consumption and emissions in various fields, such as low-carbon building construction^47^, low-carbon travel^48^, and the use of fewer household appliances^49^.

## Conclusions

This paper analysed the land cover changes in Shandong Province from 2000 to 2020 using the GlobeLand30 land cover maps. The spatial and temporal distribution of carbon stocks influenced by land cover changes was obtained according to the changes in land cover types. Carbon sources and sinks were studied in conjunction with annual average NDVI data and annual average NEP data. Land use intensity changes and anthropogenic carbon emissions were assessed based on statistical yearbook data. The specific conclusions are as follows.

1. The main change in land cover in Shandong province during the period 2000–2020 was the conversion from cultivated land to artificial surfaces. From 2000 to 2010, a total of 7748.14 km^2^ of cultivated land was transferred, with 4930.62 km^2^ being converted to artificial surfaces. Between 2010 and 2020, the proportion of cultivated land converted to artificial surfaces was as high as 78.35%.
2. The total carbon stock of vegetation influenced by land cover change decreased by 463.96 × 10^4^ t and 193.50 × 10^4^ t between 2000–2010 and 2010–2020 respectively. From 2000–2010, 164.59 × 10^4^ t and 469.44 × 10^4^ t of vegetation carbon storage were lost from cultivated land and forest to other land types, respectively. A total of 105.65 × 10^4^ t of vegetation carbon storage was lost from the conversion of other land types to water bodies between 2000 and 2020.
3. In 2000, carbon sources in Shandong Province were relatively widely distributed, mainly in the eastern coastal areas of Shandong Province, and carbon sinks were mainly in Zibo City, Jinan City, Jining City and Heze City. In 2019, carbon sources were mainly in the south-central region of Shandong Province, and carbon sinks were mainly in the western and southwestern regions of Shandong Province.
4. Land use intensity changes in Shandong province showed an increasing trend from 2000 to 2020. GDP increased from 8278.06 × 10^8^ Yuan to 71,067.53 × 10^8^ Yuan. In particular, industrial output value and construction output value increased by 4.27 times and 4.57 times respectively between 2000 and 2010. Total energy consumption in industry was the largest in terms of total energy production, closely followed by total energy consumption in transportation, storage and postal services. Most other sectors showed a more pronounced upward trend in energy consumption.
5. The conversion of cultivated land to artificial surfaces and forest to cultivated and grassland in Shandong Province, influenced by population growth and the expansion of construction land, are the main reasons for the decrease in vegetation carbon storage. Jining City is an area where the conversion of water bodies and cultivated land is more apparent, related to the construction of water storage facilities, river ditches and aquaculture ponds, and also linked to the results of remote sensing image interpretation. The conversion of water bodies and wetland in Dongying City is related to policy factors.

This study provides theoretical support and basic data for various land management policies of local governments from a more holistic perspective. In order to achieve the goal of carbon neutrality, we should slow down the expansion of artificial surfaces and increase land with higher biomass (e.g., forest), which in turn improves the vegetation carbon storage capacity of the land. Relevant government departments should promulgate and implement relevant policies on energy conservation and emission reduction, optimise the land use structure and consider reducing carbon emissions while ensuring economic development.

## Data Availability

No datasets were generated or analysed during the current study.
